# Characterizing the Digital Health Citizen: Mixed-Methods Study Deriving a New Typology

**DOI:** 10.2196/11279

**Published:** 2019-03-05

**Authors:** John Powell, Ulrike Deetjen

**Affiliations:** 1 Nuffield Department of Primary Care Health Sciences University of Oxford Oxford United Kingdom; 2 Oxford Internet Institute University of Oxford Oxford United Kingdom

**Keywords:** eHealth, health information seeking, perceived health, health service use, health outcomes, digital divide, digital inequalities

## Abstract

**Background:**

A key challenge for health systems harnessing digital tools and services is that of digital inclusion. Typically, digital inequalities are conceptualized in relation to unequal access or usage. However, these differences do not fully explain differences in health behavior as a result of health-related internet use.

**Objective:**

Our objective was to derive a new typology of health internet users based on their antecedent motivations and enablers, to explain how individuals’ different orientations influence their health behavior.

**Methods:**

We used a mixed-methods design using (1) qualitative data from 43 semistructured interviews about individuals’ general and health-related internet use, and how this influenced their health perception and their help-seeking decisions, and (2) quantitative data from the Oxford Internet Surveys (OxIS), a household survey of 2150 adults in England about their internet use and other characteristics. We used the interview data to identify constructs that described motivations and enablers affecting how internet use shaped respondents’ health perception and health service use. We then used these constructs to identify variables in OxIS, which provided a quantitative measure of these constructs. We then undertook a hierarchical cluster analysis of these constructs, using the numerical variables, to derive a proposed typology of health information seekers.

**Results:**

Both the qualitative findings and the subsequent cluster analysis suggested the existence of 6 types of individuals, categorized as learners, pragmatists, skeptics, worriers, delegators, and adigitals. Learners had a strong desire to understand health better. They used the internet to make decisions about whether they needed to see a professional and to learn about their and others’ health. Pragmatists primarily used the internet to decide whether seeing a doctor was worthwhile. Skeptics were skeptical of physicians and the medical system and valued the internet for solving health problems that doctors may not be able to deal with. Worriers found it difficult to interpret health information online, described health information seeking online as frightening, and reported a critical attitude toward online health information despite seeking it frequently. Delegators comprised nonusers and users valuing the internet as an information source, but not necessarily wanting or being able to use the internet themselves. Adigitals comprised many nonusers, but also users, who did not see the internet as a useful information tool and presented strong views on its low suitability for health care.

**Conclusions:**

This research supports a shift in the understanding of the digital divide in health, away from only access and usage issues, toward also conceptualizing an outcomes divide, whereby different types of health behavior result from the differing orientations of internet users accessing online health information. This new typology can be used to inform digital inclusion policies in health systems.

## Introduction

### Background

Health systems are under increasing pressure to save money and improve quality [1]. There are hopes that over the next decade, digital health, and specifically the internet harnessed as a health service tool, can address these aims by shaping individuals’ service use and their health perceptions. As new digital tools and new models of health service provision emerge, there are implicit assumptions that health consumers will take on new roles and responsibilities as digital health citizens [2].

In this brave new world of technology-enhanced health care, where digital technology is increasingly becoming a determinant of health, a key challenge is that of digital inclusion. Even though the digital divide in terms of internet access may have been reduced with rising internet penetration, inequalities remain in the ability to make meaningful use of online resources and to obtain benefits from doing so [3,4]. Achieving health outcomes depends on enablers of internet skills [5-7], particularly in terms of making sense of information quantity and quality [8], and health literacy, in the sense of being able to translate the findings into health-promoting behaviors [9-11]. In addition, motivations differ: not everybody can and wants to assume responsibility for their health [12,13]. Differences in outcomes are also reflected in the general shift toward referring to digital inequalities rather than to a single digital divide [14,15], and to distinguishing between the *access divide* around the turn of the millennium, the *usage divide* over the last 10 years [3], and, more recently, a third-level digital divide in relation to the benefits that individuals obtain online [4,16].

### Objective

Following these developments, this research drew on empirical data to propose a new typology of individuals’ orientations toward online health information. These orientations shape how individuals use the internet in the health context in the first place, but also, as we argue in this paper, they can explain individuals’ differing health behaviors that occur as a consequence [17,18] in terms of their health perceptions and health service use (with implications for health outcomes). We propose the *outcomes divide* as a conceptualization of digital inequalities in the context of health outcomes: that is, even accounting for differences in access and usage, there remain inequalities in the health outcomes achieved through the use of digital tools by these different types of individuals.

We derived this typology by combining findings from both qualitative and quantitative work. We did this by identifying antecedent factors in individuals’ motivations (their attitudes and desire to use health information from online sources) and enablers (their ability and interest in accessing the internet, and making sense of the information found). This is in line with various studies that found that antecedent factors must be incorporated when analyzing internet use [19,20], in particular with respect to individuals’ motivations and enablers [3]. Motivations and enablers form a complex relationship with how individuals obtain outcomes online: attitudes, awareness of technology, desire for information, job requirements, skills, and social contacts shape how individuals use technology and what they need to get out of it—and their health behaviors and subsequent outcomes in turn shape their future expectations of use [17,18].

## Methods

### Research Design

We undertook a mixed-methods research design using both face-to-face interviews and quantitative analysis of a survey dataset, as part of a larger study about the relationship between internet use and health outcomes. We conducted 43 face-to-face interviews about individuals’ general and health-related internet use, and how this influenced their health perception and their help-seeking decisions. We recruited many of these interviewees through their participation in the survey we used for the quantitative analysis: the Oxford Internet Surveys (OxIS; 2013 report). This is a random-sample survey conducted biannually since 2003, based on randomly sampled output areas (statistical areas of about 300 individuals formed based on sociodemographic homogeneity), and then randomly selected individuals within these. Using a traditional pen-and-paper method, OxIS collects data on online and offline activities, attitudes, and skills for 2150 internet users and nonusers in England (for details of the survey methods and questions asked, see the OxIS website [21]). The Central University Research Ethics Committee of the University of Oxford approved this study (number: OII C1A 14-003).

We recruited 31 interview participants from respondents to OxIS following a 2-stage sampling process: first, we purposively selected 14 output areas from OxIS to obtain areas with diverse urban and rural characteristics and area classifications; second, we contacted all OxIS participants in these output areas between July and November 2014, and the lead author interviewed all those who agreed to participate in this follow-up research. Using former OxIS participants as interviewees for the qualitative part allowed for comparison of the qualitative and quantitative elements for single individuals. We identified an additional 12 interviewees through approaching further interviewees in public spaces of the output area (purposive sampling) and by contacting others based on recommendations of OxIS participants to include particularly information-rich cases and improve the coverage of the population (snowball sampling). As a result, the interviewees included in the final sample covered the full sociodemographic spectrum in terms of sex (there were 25 female and 18 male participants), age (range 19-85 years), educational attainment, National Statistics Socio-economic Classification (NS-SEC; from 1-4, with 1 denoting the highest socioeconomic status), and long-term health conditions.

The interviews lasted about 50 minutes on average and followed a topic guide to ask about general internet use first, then health-related internet use and specific instances of health-related use, and effects and outcomes of internet use, before concluding with final reflections, next steps, and sociodemographic information. Except for 2 interviews in which the interviewees requested to talk on the phone, all interviews were conducted in person, mainly at the individuals’ homes, and partially in public cafés. Informed consent was obtained from each participant in writing (and orally for the 2 telephone interviews). For the analysis, the interviews were audiorecorded and transcribed by 1 of the authors (UD). All interviews were manually coded by 1 of the authors (UD). Emerging codes were discussed with 2 project supervisors (the coauthor and another researcher) who also read samples of the transcripts. The coding was inductive, emerging from the data, and therefore not biased toward the topics from the quantitative data, which also allowed for unexpected themes to emerge [22].

### Analytic Approach

Our mixed-methods analytic approach was to identify constructs from the qualitative interviews that described motivations and enablers affecting how internet use shaped the interviewees’ health perception and health service use, and then to identify variables in OxIS that provided a quantitative measure of these constructs. While this approach has limitations in that we were restricted to variables already collected in the OxIS dataset, using previously collected OxIS data nevertheless had advantages in that the dataset was a well-constructed existing sample where we could link our qualitative findings to the quantitative data.

Having identified variables in the quantitative dataset that broadly matched our emergent qualitative constructs, we then derived a proposed typology of health information seekers using a cluster analysis of the numerical variables. We used hierarchical clustering, an algorithmic approach that groups individuals with similar observations into clusters based on the distance between their values. It is hierarchical, as it starts with the 2 values closest together and groups those, then continues in a stepwise fashion grouping the next closest, until all are grouped (ie, eventually, if the process is continued, there will be only 1 cluster). The underlying distance measure we used was Ward’s linkage with squared euclidean distances, since it provided better results than other hierarchical methods in general [23], and specifically for OxIS [24]. We evaluated the best fit of the clustering solution based on within- or between-cluster distance and entropy [25], and by comparing the dendrogram cluster output with the emergent findings from the qualitative data.

## Results

### Motivations and Enablers

In line with the central role of antecedent factors for understanding internet use and its outcomes, 5 main motivations for health-related internet use emerged from the qualitative interview data: convenience and speed of access at all times; preparing for appointments; “translating” health professionals’ advice through nonmedical terminology online; building up further health-related knowledge; and connecting with others to get peer advice. The qualitative findings also suggested that these enablers could be traced back to 4 interrelated prerequisites for using the internet: devices to connect to the internet; skills influencing ability to read and use online information (including both technical skills and health literacy); interest in using the internet; and appropriate opportunities for use.

Having identified these influence factors from the qualitative interview work, we mapped these onto variables that had been measured in the OxIS data, creating 8 constructs for which we had quantitative data (the mapping was not perfect, as discussed in the Limitations section below). First, constructs relating to *internet usefulness* *—* the internet being an efficient means of finding information, making life easier and helping to save time—reflect the internet’s convenience. Second, the motivation relating to interpreting and extending professional advice and building up further knowledge is encapsulated in individuals’ *learning attitudes*. Third, the motivations for building up further knowledge also reflects a certain level of *online enjoyment*: enjoying reading and understanding all about certain topics online. Fourth, people’s attitudes toward medical professionals were revealed in the motivation to check on the doctor, for which the quantitative concept of *trust in medical doctors* may be an acceptable reflection.

With respect to enablers, fifth, self-rated *internet skills* conceptualize the skills dimension. We recognize that it would additionally have been desirable to include a specific measure of health literacy (as separate from technical skills) to account for the ability to find and carefully interpret medical information online [26]; however, this was not available in the OxIS data. Sixth, for the attitudinal aspect captured in the dimensions of interest and usage opportunities, *internet interest* reflects an individual’s desire to access and use the internet. Seventh, *technology attitude*, about how individuals view the general upsides and downsides of technology, relates to the wider attitudinal aspects in relation to enablers (especially “interest in using the internet” from our qualitative work). Eighth, we included the OxIS variable for *self-efficacy*, capturing to what extent individuals consider themselves as actively shaping their health, because this theme surfaced in several of our interviews and also reflects the extant health behavior literature [27].

### A Typology of Health Information Seekers

**Figure 1:**
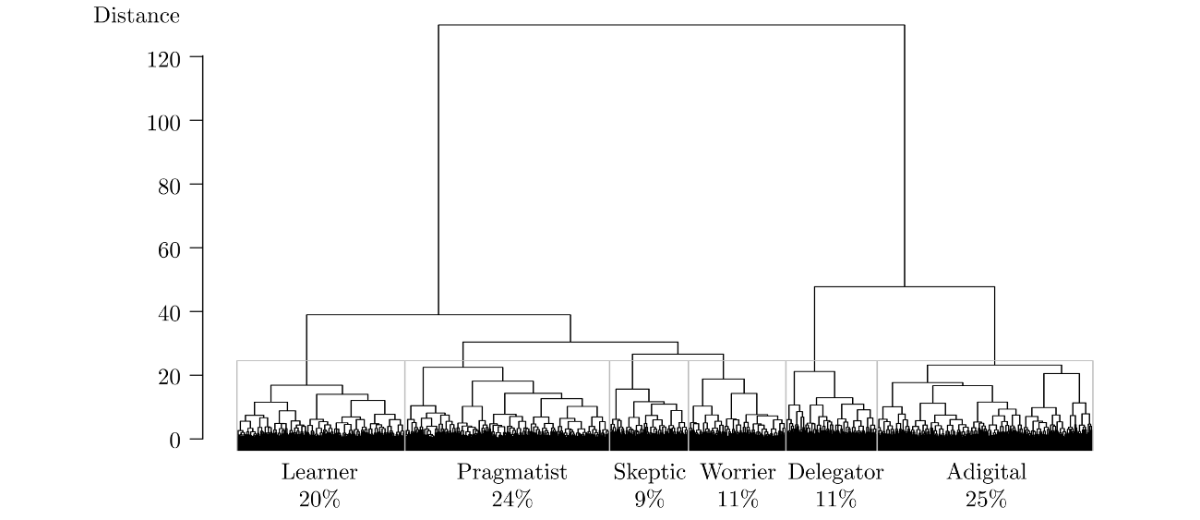
Dendrogram for hierarchical clustering of typology. Percentages are from weighted Oxford Internet Surveys data.

Having identified constructs qualitatively, and where possible identified quantitative variables from the OxIS data that measured these, our next step was to conduct the cluster analysis using OxIS. On the basis of the dendrogram shown in Figure 1, we could have justified different numbers of groups, but we chose 6 clusters not only as being supported by the cluster analysis, but also because this reflected (and supported) the qualitative findings because, over the course of the interviews, the emergent findings had indicated that individuals could be grouped into 6 different types in terms of their motivations and enablers.

The average within-cluster distance was smaller (2.5) than the between-cluster distance (4.3), with an entropy value of 1.7, so that the quality of the solution was in the range of other cluster models [25].

**Figure 2:**
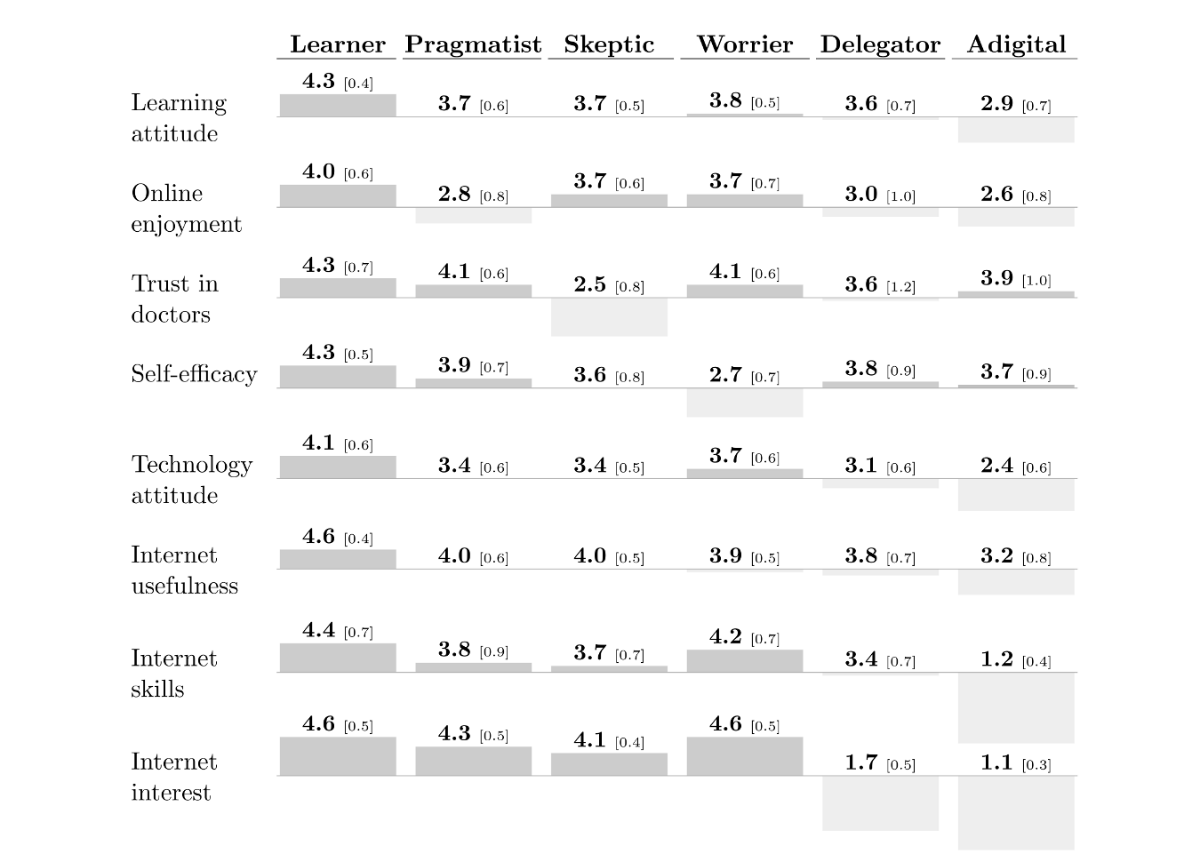
Typology of health information seekers showing cluster dimensions. All values are mean [SD]. The diagram shows the divergence from the arithmetic mean for each of the clustering dimensions. All constructs are measured on 5-item Likert scales.

Based on the distribution of the constructs within each type, we named the 6 types *the learners*, *the pragmatists*, *the skeptics*, *the worriers*, *the delegators*, and *the adigitals* (Figure 2). Despite a user-nonuser split visible in the dendrogram, these types cut across users and nonusers: 0.7% (3/422) to 4.1% (10/245) of individuals in the first 4 types were nonusers (of whom nearly all cited a lack of devices or skills as their main reason for nonuse), while 28.4% (65/229) of the delegators and 90.6% (491/542) of the adigitals did not use the internet. All groups included those with long-term health conditions (between 50/514, 9.7% and 46/229, 20.0% across the first 5 types), particularly the adigitals (262/542, 48.3%). The frequency of health information seeking also differed, with the worriers looking up health information most frequently (µ=1.8 on a 5-item Likert scale).

**Table 1:** Logistic regression for types of health information seekers(N=2150; largest condition index=4).

Independent variables	Types of health information seekers											
	Learner		Pragmatist		Skeptic		Worrier		Delegator		Adigital	
	Odds ratio	*P* value	Odds ratio	*P* value	Odds ratio	*P* value	Odds ratio	*P* value	Odds ratio	*P* value	Odds ratio	*P* value
Learning attitude	1.83	<.001	0.94	.50	0.55	<.001	0.51	<.001	1.44	.049	0.15	<.001
Online enjoyment	5.32	<.001	0.45	<.001	1.07	.58	1.13	.33	1.35	.09	0.13	<.001
Trust in doctors	2.76	<.001	1.21	.009	0.10	<.001	3.80	<.001	0.97	.80	0.72	.22
Self-efficacy	5.14	<.001	3.02	<.001	0.44	<.001	0.05	<.001	0.81	.13	1.14	.70
Technology attitude	1.86	<.001	0.54	<.001	0.56	.001	0.79	.21	0.82	.34	0.58	.31
Internet usefulness	1.80	<.001	0.71	<.001	0.43	<.001	2.70	<.001	2.68	<.001	0.30	.003
Internet skills	3.99	<.001	0.60	<.001	3.04	<.001	0.55	.01	73.69	<.001	0.00	<.001
Internet interest	4.14	<.001	17.98	<.001	14.15	<.001	20.03	<.001	0.00	<.001	0.00	<.001
Age^a^	1.04	.76	1.15	.11	1.18	.27	0.88	.37	0.59	.004	0.82	.70
Sex^b^	1.06	.52	1.00	.98	1.06	.61	1.90	.34	1.25	.11	0.94	.86
Education^c^	0.88	.25	1.17	.06	1.09	.498	0.94	.65	0.98	.92	0.86	.68
NS-SEC^d^	1.04	.73	0.98	.75	1.10	.43	0.98	.91	0.83	.25	0.78	.54
Long-term health condition^e^	0.92	.49	0.90	.19	0.92	.55	0.93	.56	0.78	.08	0.95	.87
Pseudo-R^2f^	0.73	—^g^	0.48	—	0.62	—	0.71	—	0.77	—	0.98	—

^a^Measured as a continuous variable (in years).

^b^Binary (male or female).

^c^The highest level of qualifications attained (none, primary, secondary, further, higher).

^d^1 of 5 categories in the National Statistics Socioeconomic Classification (NE-SEC; professional, intermediate, manual, unemployed, student)

^e^Binary (yes or no).

^f^Following Cragg and Uhler’s definition.

^g^Not applicable.

To delineate the types using the clustering dimensions and further sociodemographic characteristics, Table 1 presents a logistic regression on each type. It shows that sociodemographic features are less informative for determining individuals’ types and illustrates that individuals are not deterministically assigned a type, but rather have a higher probability of falling into one type or another based on their antecedent factors.

Finally, we returned to the qualitative interview data, both to confirm the validity of the clusters (that they were indeed reflected in the qualitative findings) and to illustrate the types by describing a representative of each one in more detail, as follows (real names have been replaced with pseudonyms).

The *learners* had a strong desire to understand health better. They used the internet to make decisions about whether they needed to see a professional and to learn about their and others’ health. Miriam (age 58 years, NS-SEC class 2, education level 2 of 4), who had had a minor stroke and arthritis, is an example of this group. Before and after seeing the doctor, she usually read everything she could find, in line with the generally high learning attitude of the learners (“The doctors give you the basic information that you need to know, this is what it’s called, [but] it explains a lot more on the internet.”). Consequently, she also reduced her health service use (“I still do go back to that and rather than go to the doctors and say, well this has happened…I would go on there and reread through it.” *)*. Her high level of online enjoyment and skills was also reflected in Miriam’s enjoyment in looking up health information, also for other people’s conditions (“I do like to read up on these, so I see if there’s something I haven’t got, just see how people...deal with a situation”). Miriam also set herself up as a lay expert for other people in her social circle (“If somebody’s brought something up that something’s happened to them, I tell them that I would give them my point of view once I’ve read up on it on the internet.”).

The *pragmatists* primarily used the internet to decide whether it was worth seeing a doctor. For example, Hugh (age 45 years, NS-SEC class 1, education level 4) primarily wanted to make quick decisions about health service use and the required urgency, with a low level of enjoyment (“I would use it just to kind of get a handle on whether it’s worth going to the doctor or not, not for kind of detailed self-diagnosis.”). Hugh also showed high skills and high trust in doctors combined with professional respect (“I might challenge [the doctor], but I let him...give his diagnosis first.…You have to rely on the expertise of the professional first.”). Like other pragmatists, Hugh did not want to share and discuss health problems online but valued official information and online health services (“I wouldn’t be any more concerned about privacy or security than with internet banking.”). Like Hugh, many pragmatists showed a high understanding of the need for new approaches to health provision (“I’m all for putting less pressure on the health service through faster [and] more efficient forms of medical support...[but] in Britain people are very proud of their health service...[it’s] sacred ground.”).

In contrast, the *skeptics* are skeptical of physicians and the medical system and value the internet for solving health problems that doctors may not be able to deal with, as Brian (age 53 years, NS-SEC class 1, education level 3) openly showed (“The doctors are more and more useless now as time goes on…[online] there’s forums and you can cross-reference things a bit better, rather than [depend on] the opinion of one person like the GP.”), also due to the availability of user-written information. Brian thought the internet reduced his health service use overall *(* “Probably if I didn’t have the information via the internet, maybe I would need to go and see the doctor more often.” *)*, although he also provided examples of increased health service use *(* “I have high blood pressure, and [the doctor] said that’s nothing to worry about.…So I took my own blood pressure readings...went back to the doctor, and he said, okay, I’ll give you some blood pressure tablets.”). In general, the skeptics mainly saw the doctor’s role as a provider of medication.

The *worriers* found it difficult to interpret health information online, describing health information seeking online as frightening and reporting a critical attitude toward it despite seeking it frequently. Helen (age 43 years, NS-SEC class 1, education level 4) had epilepsy and enjoyed browsing through health topics (“It’s not necessarily about epilepsy; it’s other things. I can spend ages on it, going on things that aren’t relevant to me, but I can also really forget most information.”). However, health information made her feel afraid (“I’m afraid of what I might find. If you’re on your own and look at a website, and find something really bad, [it’s] really dangerous.”). In line with the low self-efficacy of this group, Helen did not proactively want to address epilepsy (“I haven’t brought up [my children’s potential epilepsy] with my doctors. Because I think I’m afraid to do it. And I haven’t looked on websites because it’s very personal.”). She exhibited a high level of trust in her doctors (“It’s really important that you work with your doctors and your specialist and not go on the website, because it could really make it worse.”), showed a strong normative attitude about health information online (“I only really look up official websites...I’ve always been told by people not to look up health care...because you always see the horror stories”), and said that she would value recommendations about specific websites from her doctors *.*

The *delegators* were composed of nonusers and users valuing the internet as an information source, but not necessarily wanting or being able to use the internet themselves. Kathleen (age 75 years, NS-SEC class 1, education level 0) had elaborate networks to access health information (“I can go and get it from the library....If I really do want more, I’ve got a friend in London who’s got a computer, and she would...phone me back and tell me or she’ll send it down to me.”) and valued the comfort of doing so (“I don’t know whether I really do need to have a computer, because anything like that I can find out.”). Kathleen also actively read health information in the local newspaper, showing her interest in health and feeling responsible for her well-being. While trust in doctors differed among delegators, as evidenced by the high standard deviation in this group, Kathleen placed high trust in her doctor, and—illustrating again the delegation aspect—valued that he followed up newspaper articles that she took into the consultation (“He doesn’t just do it like from what it says in the paper...but takes it home and googles it on his computer.”).

The *adigitals* comprised many nonusers, but also users, who did not see the internet as a useful information tool and presented strong views on its low suitability for health care. Charlotte (age 78 years, NS-SEC class 2, education level 0) was a nonuser and generally did not like to work with technology, also due to health-related reasons (“My fingers have never worked in a way that I can use a keyboard of any sort, piano, computer keyboard, I can’t separate them. So I lost patience.”). For health information, she either would ask the doctor or, above all, thought that she knew what was best for her (“In most ways you know your own body....[I follow] just my own instincts. And I found out that they never let me down, fortunately.”). While the adigitals did not show a consistent picture of trust in medical professionals, they generally expressed concerns about how people use the internet for health information (“They’ll worry themselves into goodness knows what and they do the same on the computer—as soon as she sneezes she looks it up on the computer.”).

## Discussion

### Principal Findings

In line with the shifting digital divide from the *access* to the *usage* divide [3,15], this research supports a further shift to conceptualizing an *outcomes* divide or outcomes inequalities, whereby different types of health behavior result from the differing orientations of internet users accessing online health information. In contrast to the existing literature [4], the outcomes divide we propose in this paper may only partially be traced back to sociodemographic factors, as understanding outcomes requires a more nuanced view not necessarily following the user characteristics underlying the several existing digital divide conceptualizations [28]. This is not to downplay the importance of sociodemographic factors and other structural conditions that can shape internet use. For example, previous work has shown the strong influence of socioeconomic status on internet-related attitudes and behaviors [29]. But in our findings these factors, while important at an overarching level, did not have different influences on our various categorization of types, where we were particularly interested in determining how motivations and enablers clustered into types.

This study showed the central role of antecedent factors to internet use for influencing behaviors. The qualitative data confirmed motivations [30,31] and enablers [32] found in previous research, and indicated that outcomes may be shaped by types of health information seekers formed based on these motivations and enablers. This confirms that previously existing health behaviors translate to the online realm [29,30,33], and emphasizes attitudes and skills as mediators for internet outcomes [17]. In that sense, online health resources become part of normal health practices, help seeking, and everyday life information seeking [34-36]. This ties in with the wider argument that individuals use technologies to satisfy existing needs, with technological innovation merely creating new ways of doing so [37], as reflected in theories about the social shaping of technology [38].

The qualitative data suggested that 2 of our 6 types, the learners and the pragmatists, use the internet efficiently in health-related contexts, both to increase the appropriateness of their health service use and, in the case of the learners, to gain self-efficacy for self-care and extending professional advice. In that sense, individuals in both groups consistently gain benefits from using the internet.

Then again, outcomes do not necessarily have to be positive. For the skeptics, the suggested relationship to perceived health was negative, which may indicate that using the internet was less beneficial than the skeptics thought. In support of this, other research found that low-trust individuals tended to substitute physician services with health information online [39,40], and while those skeptical of medical care had lower health service use, they also often showed worse health behaviors and lower health perception [41,42]. For the worriers, internet use was barely associated with any changes in perceived health and health service use. While health information seeking does not necessarily lead to higher health service use and worse health perceptions, the findings indicated that this group did not necessarily realize any outcomes, partially because they stopped looking up health information as a consequence.

Finally, the effects of internet use were lowest for the delegators and the adigitals, although with higher effect sizes for the delegators. Some nonusers in the delegator group used the internet more intensely (via intermediaries) than users, building support networks with different individuals for different purposes. While both the delegators and the adigitals largely comprised nonusers, this shows how internet outcomes may not follow the lines of the user-nonuser split. This is further corroborated by the relatively similar outcomes for users and nonusers in the delegator group, which may partially be due to their preference for outsourcing health-related information seeking.

The typology introduced in this research therefore presents a tool for systematizing orientations toward health information seeking to conceptualize the outcomes divide. This is similar to other typologies in research on health and internet use [43,44], which serve to “shift study of the Internet away from an overly narrow focus on comparing users and non-users, and [focus] more research and debate on other variations among users and non-users that have equally significant implications for the future of the Internet” [24] (pg 9).

### Limitations

A limitation of our approach is that, for 6 of the 31 OxIS interviewees, the quantitative and qualitative classifications did not correspond, so that we manually reclassified these based on the qualitative data. There are three reasons for this mismatch. First, in line with the process-based models of perceived health [45], individuals may transcend the type boundaries over time, which became evident in 2 interviewees’ altered views of the health system after major health incidences. Second, the typology lacks some specificity due to the absence of some items in the OxIS question set. These include the lack of a measure of health literacy, no measure of acute health incidents (only including the presence or absence of long-term health conditions), and only limited information on situational factors such as devices and use opportunities. Even though health information seeking is similar to other informational activities online [46], 2 interviewees had particular attitudes about health online that differed from their general internet views. Third, 2 interviewees showed different attitudes in the interview for no evident reason, which highlights the constructed nature of survey and interview data, and the challenges of their triangulation.

### Future Research and Implications

Further research should attempt to replicate and refine the developed typology, ideally with health-related dimensions by including more specific constructs of health literacy and health-related self-efficacy [7,47,48]. Due to transitions of individuals between types over time, the typology should also be based on longitudinal measurements on the same individuals from multiple points in time, as individuals may develop and fall into different types following different events in their lives. For the broader context of internet research, it would be relevant to understand whether the established typology also describes orientations that are relevant for other internet-based outcomes. This would cross-validate the results of this research, and thereby provide theoretical support for a more general outcomes divide beyond the health context.

These type-based findings have implications for policy and practice, particularly for health systems seeking to maximize digital inclusion. A multifaceted approach is required to address the differing needs of the 6 types. Nonusers of the learner and pragmatist type in particular should be provided with access to digital resources, as most individuals of these types cited the nonavailability of devices as the main reason for nonuse. In addition, worriers may benefit from additional guidance: they highly appreciated medical professionals and suggested that doctors should recommend specific websites reflecting their preference for professional guidance and managing uncertainty [49]. They would, for example, use initiatives that provide official endorsement or certification of digital resources and health apps. This stands in contrast to the skeptics, who—independently of the internet—might benefit from building up trust in doctors and the medical system to change their health behaviors, evaluate the appropriateness of health service use, and ensure compliance with medical recommendations [40,41].

Particularly for delegators, but also for all others with a lower level of skills, the social environment is of crucial importance for internet-based outcomes. As the type name implies, obtaining value from the internet depends on being able to delegate. Here, addressing social and digital inclusion becomes a joint priority, where not everyone has to be online, but everyone should benefit from online resources. Finally, for the adigitals, it may be most important to address motivations for internet use. This applies to users and nonusers alike: 79.8% (392/491) of nonusers of this type referred to a lack of interest as the main reason for nonuse, whereas users mainly explained why the internet was not suitable for health-related matters.

### Conclusion

This research showed how health internet users may be conceptualized based on a typology of 6 orientations toward online health information seeking. The findings illustrate that the digital divide is increasingly more complex to delineate [50], indicate that previously existing health behaviors translate to the online realm [32], and support the shift toward an outcomes divide in terms of the benefits that individuals of differing types may obtain online [4]. This research also showed that health behaviors (and, by deduction, possibly health outcomes) are primarily shaped by antecedent factors such as motivations and enablers [3,17], rather than sociodemographic factors [4]. This research therefore makes, to our knowledge, one of the first empirical contributions to an emerging literature assessing how differences in outcomes represent the next stage of continuously shifting digital inequalities.

## Abbreviations

NS-SEC: National Statistics Socio-economic Classification
OxIS: Oxford Internet Surveys
